# Emotional abuse and neglect in a clinical setting: challenges for mental health professionals

**DOI:** 10.1192/bjb.2021.90

**Published:** 2022-10

**Authors:** Margaret DeJong, Simon Wilkinson, Carmen Apostu, Danya Glaser

**Affiliations:** 1 Great Ormond Street Hospital, London, UK; 2 Great Ormond Street Hospital, London, UK; 3 East London NHS Foundation Trust, London, UK; 4 Great Ormond Street Hospital, London, UK; 5 UCL, London, UK

**Keywords:** Childhood experience, emotional abuse, emotional neglect, psychological maltreatment, adverse childhood experience

## Abstract

This article addresses some of the common uncertainties and dilemmas encountered by both adult and child mental health workers in the course of their clinical practice when dealing with cases of suspected emotional abuse or neglect (EAN) of children. We suggest ways of dealing with these according to current best practice guidelines and our own clinical experience working in the field of child maltreatment.

Within the field of child maltreatment, emotional abuse and neglect (EAN) (psychological maltreatment) refers to caregiver behaviours towards or involving a child (excluding physical/sexual abuse and physical neglect) that cause or have a strong potential to cause serious harm to all aspects of a child's well-being or development. EAN could reflect repeated caregiver acts or omissions, or a single extreme behaviour. Caregiver refers to any adult responsible for caring for the child, usually parents.

This is a form of maltreatment with a high prevalence partly because it exists both on its own and in combination with other forms of abuse or neglect. And yet it is frequently under-recognised, including by clinicians working in child and adolescent mental health services (CAMHS) and adult mental health. Part of this may be due to ambiguity and lack of consensus over the definition, and the need to distinguish between parenting difficulty and actual EAN.

Risk factors for EAN include child factors (e.g. developmental disorder), caregiver factors (e.g. psychiatric disorder and substance misuse, which will be identified by adult mental health professionals, domestic violence, parental history of childhood maltreatment)^1^ and wider family factors (e.g. large number of children, poverty).^2^

Clinicians may observe worrying interactions between parents and children amounting to EAN, or come to recognise that a referred child's difficulties are associated with such parent–child interactions. These may be formulated as contributing to the child's mental health difficulties and be a focus of treatment.

Although identification continues to be problematic, there is evidence of the detrimental impact of harmful parent–child interactions on the child's attachments and development at the time of the maltreatment, as well as later.^3^ EAN is likely to cause harm through its impact on a child's thoughts, feelings and self-concept (leading, for example, to increased susceptibility to depression); the development of maladaptive behaviours, which may serve an emotion regulation function (e.g. substance misuse); interpersonal difficulties such as relationship avoidance or aggressive behaviour; reduced educational achievement; and biological changes such as altered stress response and increased damage to health due to lifestyle factors such as smoking and risky sexual behaviour.

There is a converging weight of evidence across clinical and population samples internationally suggesting the detrimental impact of EAN on mental health outcomes. The Adverse Childhood Experiences Study asked retrospectively about experiences of EAN and several other experiences (exposure to domestic violence, sexual abuse and physical abuse) that would necessarily also involve EAN.^4^ It was found that the cumulative sum of adverse childhood experiences contributed exponentially to adverse outcomes in physical and mental health. Longitudinal studies have found a significant correlation between childhood EAN and psychotic symptoms at age 21,^5^ increased rates of depression in women at age 18,^6^ and comorbid substance misuse, depression and anxiety into the mid-30s.^7^ EAN showed the strongest association, compared with other abuse subtypes, with depression and anxiety at age 21.^8^ Few studies have been able to control for genetic risk, although one^9^ looked at emotional abuse after age 12 in 2232 English and Welsh twins interviewed at age 18, concluding that adolescent victimisation (including internet abuse) predicted psychopathology at age 18, in a pathway that appeared to be environmentally mediated.

There are, nevertheless, some problems with existing research into the effects of EAN. The widely used Childhood Trauma Questionnaire (CTQ) enquires about only one aspect of EAN, namely spurning (targeted hostility/rejection) or the general term emotional abuse.^10^ Much existing research^11^ uses the CTQ or similar, establishing in cross-sectional studies an association between a positive score for emotional abuse, using retrospective recall, and a wide range of psychopathology in adolescence and adulthood. There are some obvious methodological problems with this approach, limiting the conclusions that can be drawn. A few longitudinal studies have attempted to correct for possible bias in reporting, for example by using cases substantiated by social services, and looking at potential environmental confounders. Differences in individual susceptibility to the effects of maltreatment need to be considered, and some studies have controlled for the presence of a neurodevelopmental disorder such as attention-deficit hyperactivity disorder (ADHD). There are limited data on age at exposure to emotional abuse, as EAN is rarely one event; rather, it is an integral part of an ongoing parent–child relationship: earlier age at onset will affect key developmental processes such as attachment formation but adolescents may be more aware of being subjected to abusive or neglectful interactions, adding to the risk of harm.

Clinicians suspecting the existence of emotional abuse or neglect in the parents and children they see are faced with a number of dilemmas. We attempt to identify these and offer current best practice solutions in the hope that this will improve awareness of and confidence in managing these cases.

## Questions and dilemmas

### Are there recognised patterns of interaction in emotional abuse?

In part as a response to difficulties in defining emotional abuse, clusters or categories of commonly observed interactions have been described both in the USA^2,12^ and in the UK.^13,14^ Several categories may occur together. They are designed to help assessing clinicians and to provide guidance towards the focus of treatment:

1. emotional unavailability, unresponsiveness and neglect
2. hostility, blame, denigration, rejection or scapegoating
3. developmentally inappropriate or inconsistent interactions with a child
4. exposure to frightening or distressing experiences
5. failure to recognise a child's individuality and the psychological boundary between the parent/carer and child
6. failure to promote the child's socialisation.

Most clinicians will easily recognise categories 1 and 2. In a CAMHS setting, category 1 may take the form of a parent/carer's lack of support for recommended treatment/psychosocial interventions for their child.

Category 3 includes, as examples, harsh and inconsistent discipline, inappropriately high expectations of a child that are well beyond their developmental level (such as doing excessive housework or taking on a caring role), but equally cases of overprotective behaviour, usually stemming from a parent's own anxieties. These interactions are styles of harmful parenting, rather than interactions targeted at an individual child.

Category 4 includes inappropriate exposure to disturbing or traumatic events such as occurs in domestic violence. It will also present as a concern in adult mental health services, where some children of mentally disordered adult patients will be recognised as observing highly distressing or threatening behaviour in their parents.

Category 5 is seen, for example, in fabricated or induced illness, where a parent's own needs to have a child perceived as ill outweigh the child's needs for nurturing of their separate, individual identity. The psychological boundary between parent and child becomes blurred. It can also be seen in couple disputes, where children are used as pawns in marital conflict and breakdown, sometimes confided in inappropriately and drawn in by one parent to take sides against the other parent. At its most extreme, children can be pressured to make or collude with false allegations against a parent. Another example may be seen in parents who have experienced traumatic events or abusive relationships and wish to protect their child from similar experiences but in doing so do not accurately distinguish between their own psychological realities and those of their child.

Examples of category 6 include parents who misuse substances, who expose their children to practices such as shoplifting to fund a drug habit, or families who for a variety of reasons fail to support their child's attendance at school and isolate their children.

Helpful as these categories are, not all observed clinical cases will fit neatly, although most tend to have a predominant theme in at least one.

### What to do if there are concerns about a parent–child interaction and EAN is suspected

The child is likely to have been referred with difficulties, which should be carefully noted. The next most important action is to carefully record clinical observations, including of parent–child interactions and child behaviour and reactions. Accurate record-keeping is essential in any potential child maltreatment situation, but particularly important in EAN, where it is the repetitive nature of negative interactions that is so damaging. Making a case of emotional abuse often relies on descriptions of repeated, consistent observations, in different settings and ideally by different observers. Descriptions will also be useful in most instances to raise the concern with the parent (without naming emotional abuse) to see whether they are able to acknowledge unhelpful or harmful interactions with their child.

The clinician may at this stage wish to discuss with colleagues, or simply arrange to see the family again, in order to verify the observations and gauge the parental response. If the pattern is repeatedly observed, links need to be assessed between the child's difficulties and the forms of the parent–child interactions of concern.

There are a few rare situations in which emotional abuse is associated with immediate serious risk to the child. An example might be abandonment of a child or a parent experiencing a psychotic episode whose child is caught up in the parent's delusional beliefs. If the child is aware, they may be confused and troubled, but more importantly in extremely delusional states the child's life may be at risk.

### How to tell the difference between family dysfunction or parenting difficulties and abusive parenting

Clinicians will be used to working with families in which there are challenging dynamics and communications, or supporting parents whose parenting may be adversely affected by factors such as poverty, mental ill health and their own adverse history of being parented. The distinction between parenting difficulty and abuse needs to be made with reference to the definition of EAN, which is the presence of persistently harmful interactions or a single severely harmful incident with actual or likely harm to the child.

### What does a good clinical assessment of emotional abuse look like? What should it include?

This again goes back to the definition of EAN, which includes evidence of harmful parent–child interactions that can be linked with some certainty to evidence of harm to the child's development or current functioning. The assessment begins with the child's mental state and development, including cognitive, emotional, behavioural, social and physical functioning (the latter is important in cases of failure to thrive, feeding/eating disorders or medically unexplained symptoms). Collateral information is especially important here and the school can usually provide invaluable information, including their observations of parent–child interaction.

It is particularly important to consider neurodevelopmental disorders and other innate child vulnerabilities that might increase a child's susceptibility to the effects of problematic parenting or might fully explain the child's difficulties. Children with neurodevelopmental disorders are at increased risk of experiencing abusive parenting;^15^ one likely reason for this is the more demanding nature of looking after a child with, for example, ADHD, autism spectrum disorder or an intellectual difficulty. Children with a physical disability are also at increased risk.

Detailed assessment of the specific parent–child interaction of concern follows. An overall parenting assessment must be nuanced and comprehensive, to include parenting strengths relevant to any observations.

The assessment is then extended towards the parents, including mental state and functioning, their family history and family relationships. Risk factors include parental mental ill health, substance misuse and domestic violence occurring as isolated risk factors or in combination.^16^ Parents’ own experiences of poor parenting, including disrupted attachments, especially intergenerational involvement of child protection agencies, are a cause for concern. However, it must be stressed that most adults with mental health difficulties do not abuse their children and many adults with disadvantaged backgrounds go on to parent successfully.

Social stressors such as unemployment, poverty, social isolation, community violence and criminality are likely to have an impact on parenting.

### How can I tell whether a child's symptoms are due to EAN and not something else?

The impact of EAN on children is mostly non-specific and it can be part of the aetiology of a very wide range of psychological symptoms and impairment in the child. The assessment requires a detailed knowledge of child development and also an in-depth knowledge of the particular child being assessed. A careful process of exclusion must take place to be sure that an innate developmental disorder is not a significant factor. If a neurodevelopmental disorder is found to be present, does it fully explain the child's difficulties? It is of course possible for EAN to be an additional contributing factor in a child with ADHD or autism spectrum disorder. Aspects of the child's internal world, such as self-concept and expectations of others, are relevant to the assessment. The clinician looks out for evidence of a link between negative self-concept or unhappiness and observed negative parent–child interaction. The degree to which EAN is thought to be a significant aetiological factor in a child's difficulties is a matter of clinical judgement, but based on a careful, thorough and open-minded assessment.

EAN may have significant negative impact without a child reaching the threshold for a diagnosis. Subthreshold presentations or negative impact on general psychological functioning or development are also important and a child may engage in very risky behaviour, such as running away from home or becoming sexually vulnerable, without meeting criteria for a diagnosis. A child may seem resilient, for example being academically bright and able to make use of relationships with other adults, but still be experiencing harm in terms of gradual impact on self-concept and behaviour due to repeated negative interactions that undermine emotional development and other aspects of functioning. This is a form of hidden harm, which wears away resilience and may surface as a concern at a later date. The EAN definition includes ‘potential for harm’, which is relevant here. Based on knowledge of the child and child development it is possible to predict that if EAN continues unchecked the detrimental impact on the child will become more evident over time.

### How do we allow for differing cultural practices?

Informing oneself about a culture is an important first step, sometimes requiring a consultation with someone very familiar with the particular family's culture. However, certain cultural practices relevant to emotional abuse are not condoned in the UK. An example is a belief, prevalent in a number of cultures, that an evil spirit can become lodged within a child. The child can be isolated and rejected in a way that would be described as scapegoating in an emotional abuse framework. Cultural practices therefore need to be judged within the jurisdiction's code of practice regarding child protection. The evidence of impairment of the child's development or functioning becomes particularly important in this context. Emotional abuse can occur across all cultures and socioeconomic groups.

### What is the best way to feed back concerns to parents?

In most cases feedback to parents is direct and specific, giving examples of concerning interactions and observations of the child. Feedback, given orally and also in writing, should also set out the proposed treatment and the goals of treatment. The potential for further harm to the child if parental behaviour is unchanged should be clearly set out.

### What is the best way of treating EAN?

In essence, treatment of EAN rests on addressing the relevant categories of EAN of concern. This is not to be equated with holding parents solely responsible for their children's difficulties. Clinicians should work on parents’ ability to acknowledge harmful interactions. Without acknowledgement, parents may well be less motivated to change. Different types of EAN, as depicted in the categories listed above, have been found to respond to different types of clinical approach. For example, emotional neglect is often associated with parental depression, drug misuse or social stressors such as poverty and social isolation. Therapeutic work to increase the parent's attentiveness to the child needs to follow the alleviation of these stressors. It may usefully include video feedback. Developmentally inappropriate interactions respond well to evidence-based psychoeducational parenting interventions. Failure to recognise a child's individuality or failure to promote the child's socialisation requires therapeutic work to improve ‘mentalisation’ – helping the parent to ‘put themselves in the child's shoes’. Clear hostility towards or rejection of the child are often based on a parent's belief about the child's innate negative qualities. Beliefs are difficult to shift and it is not helpful for a therapist to point out the child's good qualities. Rather, a sensitive exploration of the basis of these beliefs is required.

Some of this work will be with the parent(s) alone, but often work will be with the parents(s) and child together. The treatment of an adult's mental ill health by adult mental health services may be a core component of the treatment of the EAN. The child may have their own individual therapeutic needs but it should not be assumed that therapy for the child alone will suffice to reduce the EAN. The family may need social support. This multidisciplinary and multiagency work can only succeed with a coordinated, systemic approach. A modular approach may be needed, selecting goals for treatment in a stepwise fashion based on how pressing and achievable they are.

EAN patterns are often rigidly entrenched, and resist redirection by therapists who attempt to modify behaviour. For this reason, therapeutic work with EAN is best regarded as a time-limited trial, testing a parent's capacity to show some insight into the impact of their own behaviour, empathy towards their child and an indication of their capacity to change.

EAN is closely related to the formation of insecure attachments, and parent–child therapeutic work addresses this.^17^

Although individual work with the child is unlikely to reduce EAN, children who have experienced EAN often require therapeutic work to deal with its effects. This requires a careful assessment of the child's psychological and interpersonal functioning and needs. For instance, it is possible that enhancing the child's capacity to mentalise may reduce subsequent aggression.^11^

### At what stage should a referral to social services be made? And do we always need to inform parents?

Clinicians should seek to work collaboratively with parents in ensuring that all support needs are met. This may involve referral to early help services to access resources such as parenting programmes, family workers and assistance with problems such as housing and finances that may be contributing to parenting difficulty. Such referrals should be framed positively as being made to provide support for the family.

A children's social care referral for protection should be made when it is the clinician's judgement (having discussed it with their team or supervisor) that the threshold for emotional abuse has been reached *and* all therapeutic efforts have reached an impasse. There is a stage where therapeutic efforts are ongoing when the clinician may be reluctant to notify in case the therapeutic alliance is jeopardised. In this scenario a careful risk–benefit analysis must be considered. Once it is clear that therapy is not progressing and there is a lack of insight or acknowledgement on the part of parents, there should be no further delay in making a referral. In some cases the risk is so evident at assessment that therapy should only be carried out within a child protection framework.

In most cases parents should be informed of the referral. There are a few exceptions where there is a concern that telling parents might heighten the risk for a child. This occasionally happens in parental psychotic disorders, or where a case of fabricated or induced illness involves physical threat to a child at the hands of a parent.^18^ The risk must be discussed with the social services manager.

In the case of a referral that is declined by social services, concerns should be set out in writing to a senior manager, giving specific examples and evidence of risk to the child and escalating the concerns. A framework of EAN such as that outlined in this paper can be useful in communicating concerns.

### Once a child is formally considered at risk and is on a child protection plan, can we still proceed with therapy?

A family may be willing to engage with therapy within a child protection framework. There are well-established examples of this, for example in the fabricated or induced illness field^19^ or in work carried out within care proceedings,^20^ which we refer to as a ‘trial for change’. Therapy when there are child protection concerns is carefully constructed over a tight time frame, with clearly articulated goals reflecting those concerns. There is open liaison between the treating team and social services. It is important that the treatment goals, which should be subject to a written contract between therapist and parents, are very specific and observable in terms of the child's function or the parent's behaviour. This therapeutic work can still proceed within CAMHS, and a partnership and close liaison between health and social services teams will be the best model. In some cases the work may be carried out by clinicians employed by social services.

### What can I do if the difficulties within the family are not easily addressed by the CAMHS in which I work?

Services have limited resources and the kind of extended contact that may be required for confirming the presence of EAN (or treating it) may not fit easily into the constraints of the clinical pathways being followed. For example, a clinician may be seeing the child just for a neurodevelopmental assessment, or the child's symptoms may not meet the threshold for treatment within their service. In these situations, the child's welfare must prioritised but there may be a tension between resources available, the flexibility of available pathways and the family's needs.

In the case of neurodevelopmental assessments, it may be necessary to carry out extended assessments if there are significant concerns about parent–child interaction or to advise that a child's difficulties be re-assessed once any parent–child interaction difficulties are addressed if the clinical picture is unclear. The National Institute for Health and Care Excellence (NICE) guidelines on autism spectrum disorder in under-19s include maltreatment as a possible differential diagnosis but at the same time advise against attributing signs of possible autism spectrum disorder to a disruptive home environment, indicating the need for holistic assessment that is sensitive to both neurodevelopmental and psychosocial factors.^21^

A child may be discharged following an assessment when there are concerns about possible emotional abuse that were considered not to meet a safeguarding threshold. It is important to raise these concerns with whichever professionals have an ongoing relationship with the family, such as the general practitioner or teachers at the child's school, who are then in a position to monitor and act accordingly if these concerns do not subside. In a similar vein, if therapeutic work is being handed over to another service, then full communication of any concerns, accompanied by clear documentation, is vital.

### What is the role of adult mental health professionals?

As part of a mental health service response to EAN, a parent may also need to access their own therapy. Referral to adult mental health services will be difficult if the parent lacks insight or denies their own role. Consent issues, lack of trust and the stigma attached to mental illness are additional challenges. Issues with service structure include high thresholds for adult services, which are generally built around diagnosis and risk to self (rather than other aspects of functioning, such as parenting), and the need for a shared approach that can be hampered by issues such as confidentiality and goals of treatment differing between services. For example, a patient-centred approach to adult psychological therapy may not adequately address abusive parent–child interactions, resulting in a parent's beliefs and behaviours being unchallenged or even reinforced. For this reason, close communication is required between services to ensure a shared understanding of the safeguarding concerns and goals in relation to protecting the children. Currently, the National Health Service is not structured in a way that facilitates this much-needed liaison. Adult mental health services should enquire about the effects of an adult patient's mental health problem on their children. There have been initiatives to encourage this, such as Think Family.^22^ This would lead to improved identification of emotional abuse or neglect. Some services have benefited from innovative models such as parental mental health teams.

## Conclusions

Emotional abuse or neglect is a very common form of child maltreatment often considered difficult to recognise or define. It appears that clinical practice has not yet caught up with the growing significance attached to EAN in relation to the mental health outcomes of our patients. We have attempted to highlight here dilemmas encountered by both adult and child mental health workers in this complex area and to suggest possible ways of resolving them.

## Data Availability

Data availability is not applicable to this article as no new data were created or analysed in this study.

## About the authors

**Margaret DeJong**, MDCM, FRCPsych, is an honorary consultant child and adolescent psychiatrist at Great Ormond Street Hospital, London, UK. **Simon Wilkinson**, MBBS, MRCPsych, is a consultant child and adolescent psychiatrist at Great Ormond Street Hospital, London, UK. **Carmen Apostu**, MBBS, MRCPsych, is a consultant child and adolescent psychiatrist with East London NHS Foundation Trust, London, UK. **Danya Glaser**, MBBS, DCH, FRCPsych, Hon FRCPCH, is an honorary consultant child and adolescent psychiatrist at Great Ormond Street Hospital, and Visiting Professor, Psychoanalysis Unit, Research Department of Clinical, Educational and Health Psychology at University College London, UK.
